# Lichen planus pigmentosus

**DOI:** 10.11604/pamj.2013.15.54.2928

**Published:** 2013-06-19

**Authors:** Hayat Bourra, Benzekri Leila

**Affiliations:** 1 Dermatology Department, CHU Ibn Sina, Med V Souissi University, Rabat, Morocco

**Keywords:** Lichen planus, lichen pigmentosus

## Image in medicine

Lichen planus pigmentosus (LPP) is a rare variant of lichen planus (LP), reported in various ethnic groups. It occurs predominantly in female in the third or fourth decade of life, characterized by insidious onset of dark-brown macules in sun exposed areas and flexural folds. The differential diagnosis may occur with drug-induced pigmentation, photosensitization and vitamin deficiency like pellagra. Here we describe a case of a 51-year-old Moroccan man, working as a gardener, who presented an asymptomatic, non itching pigmented lesion on his face and neck. He had no history of trauma or medication use, and no preceding erythema or scaly skin eruption. Clinical examination revealed dark brown macules confluent at the forehead, preauricular region and temples with some papules in the neck. Oral, genital mucosa and nails were unaffected. A skin biopsy showed a lichenoide lymphohistiocytic infiltrate in the dermis with basal cell degeneration, pigmentary incontinence and dermal melanophages. The diagnosis of (LPP) was established. A hepatitis serology profile was negative. Initially, a chloroquine treatment was done but showed poor response.

**Figure 1:**
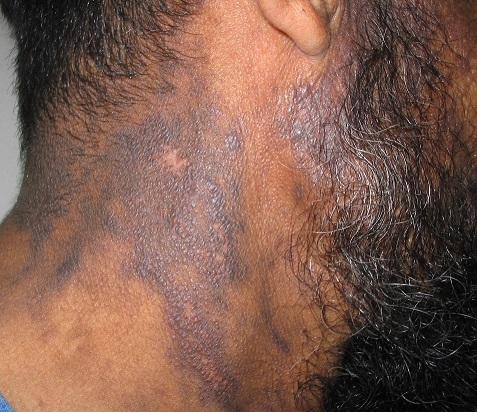
Dark brownish macules and papules of the neck

